# Supplementary figures and images for: TMEM16A chloride channel does not drive mucus production
Source: Life Sci Alliance. 2019 Nov 15;2(6):e201900462. doi: 10.26508/lsa.201900462 (PMC6859295; doi:10.26508/lsa.201900462)

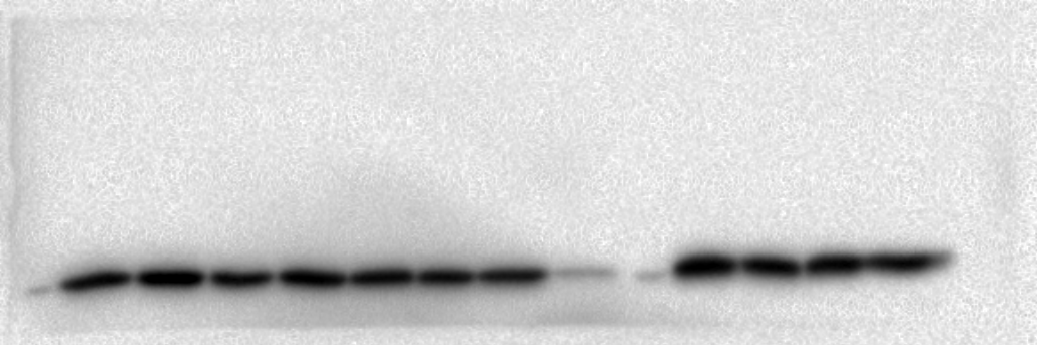

Supplement: Supplementary file 1 [file LSA-2019-00462_SdataF1.tif]

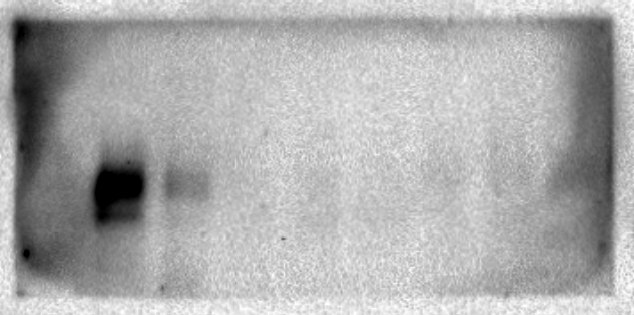

Supplement: Supplementary file 2 [file LSA-2019-00462_SdataF1.1.tif]

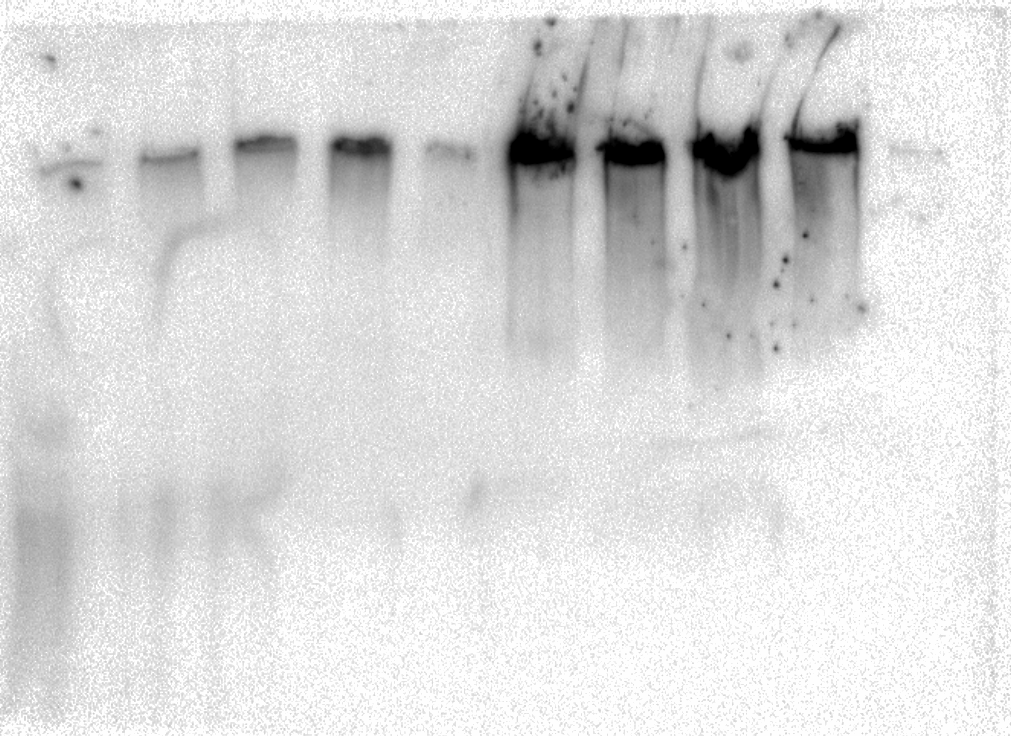

Supplement: Supplementary file 3 [file LSA-2019-00462_SdataF1.2.tif]

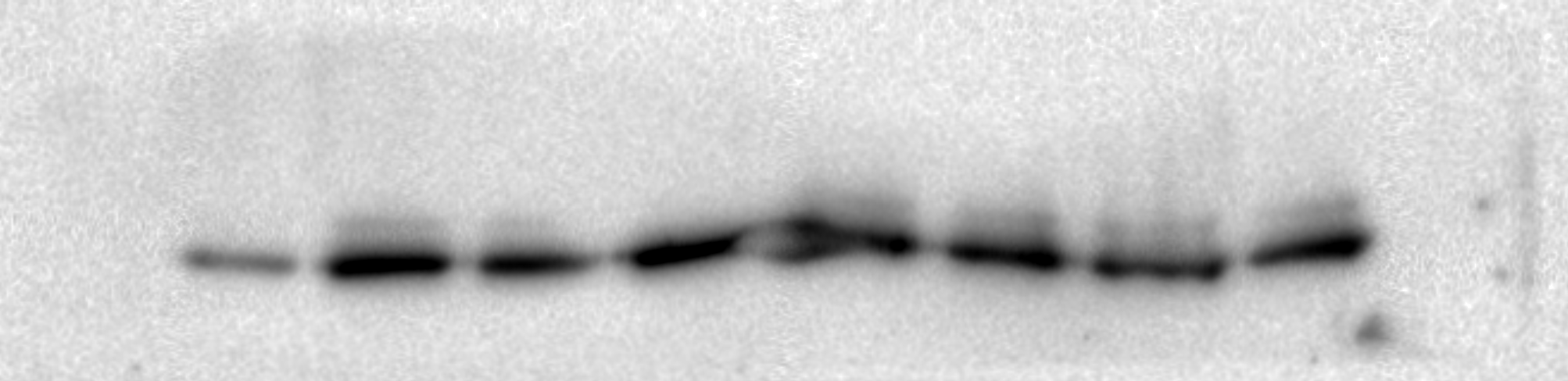

Supplement: Supplementary file 4 [file LSA-2019-00462_SdataF1.3.tif]

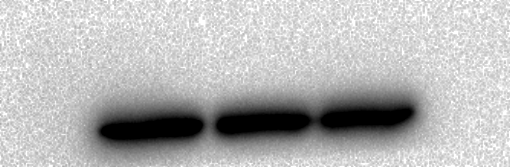

Supplement: Supplementary file 5 [file LSA-2019-00462_SdataFS1.tif]

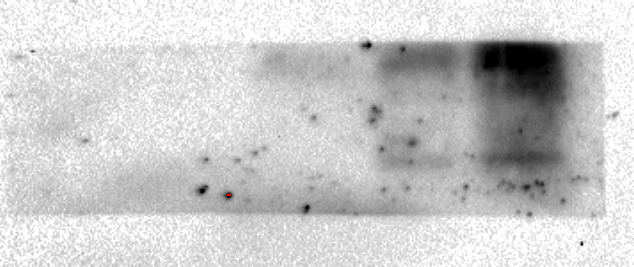

Supplement: Supplementary file 6 [file LSA-2019-00462_SdataFS1.1.tif]

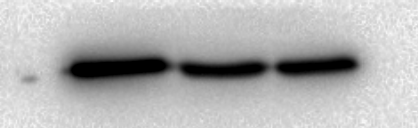

Supplement: Supplementary file 7 [file LSA-2019-00462_SdataFS1.2.tif]

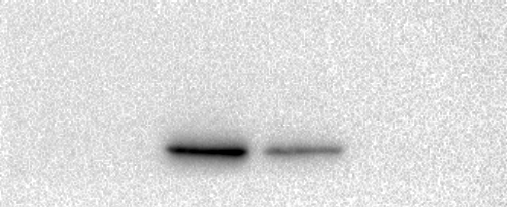

Supplement: Supplementary file 8 [file LSA-2019-00462_SdataFS1.3.tif]

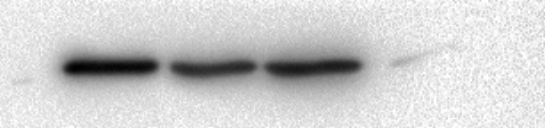

Supplement: Supplementary file 9 [file LSA-2019-00462_SdataFS1.4.tif]

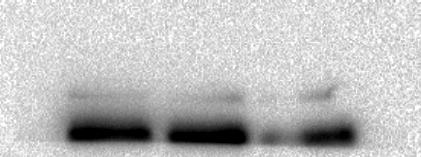

Supplement: Supplementary file 10 [file LSA-2019-00462_SdataFS1.5.tif]

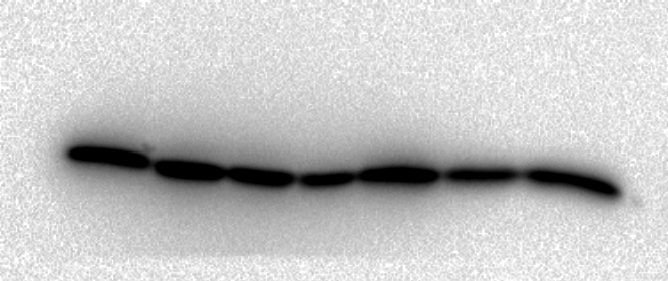

Supplement: Supplementary file 11 [file LSA-2019-00462_SdataF3.tif]

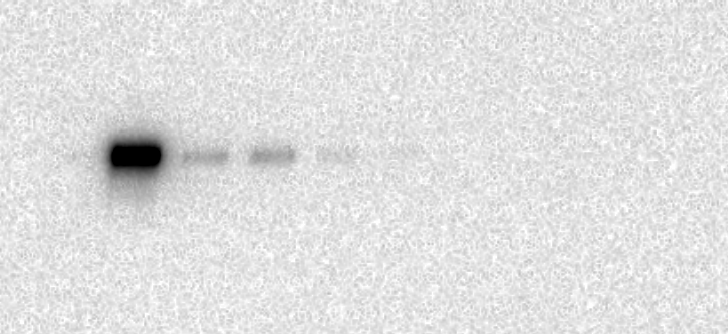

Supplement: Supplementary file 12 [file LSA-2019-00462_SdataF3.1.tif]

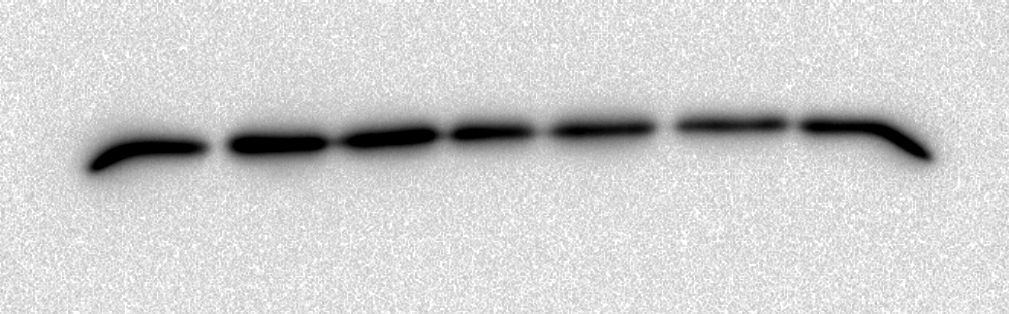

Supplement: Supplementary file 13 [file LSA-2019-00462_SdataF3.2.tif]

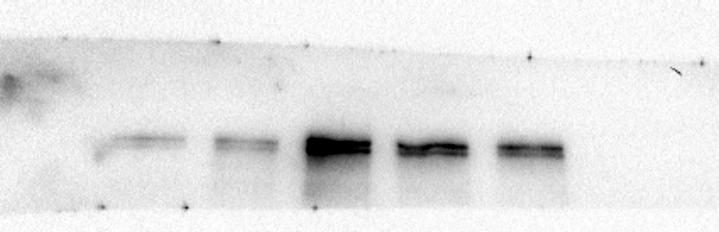

Supplement: Supplementary file 14 [file LSA-2019-00462_SdataF3.3.tif]

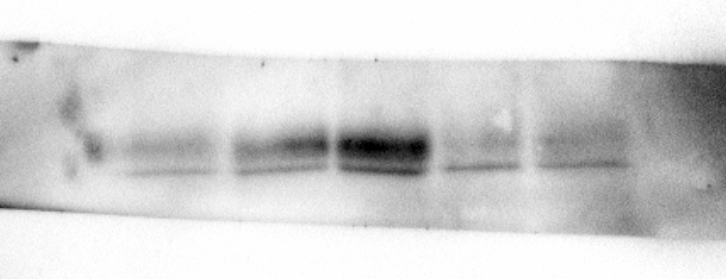

Supplement: Supplementary file 15 [file LSA-2019-00462_SdataF3.4.tif]

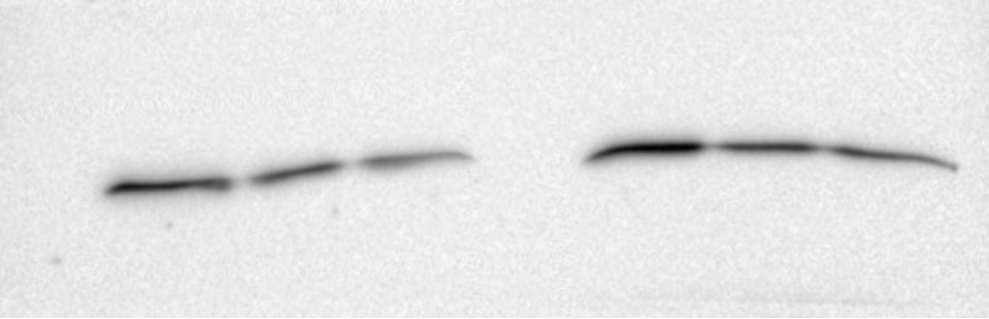

Supplement: Supplementary file 16 [file LSA-2019-00462_SdataF4.tif]

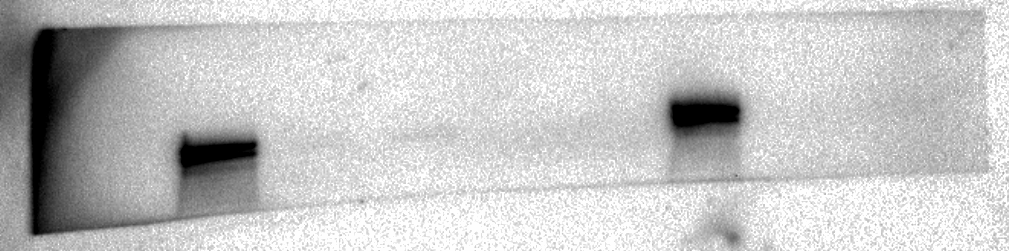

Supplement: Supplementary file 17 [file LSA-2019-00462_SdataF4.1.tif]

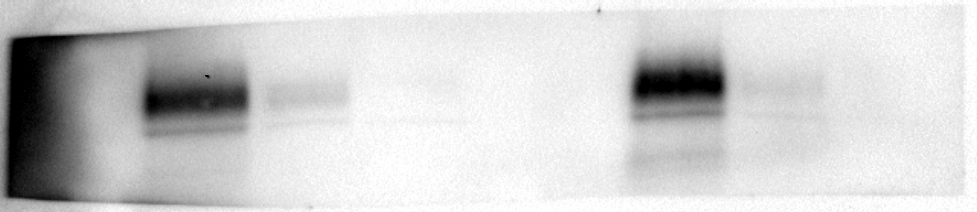

Supplement: Supplementary file 18 [file LSA-2019-00462_SdataF4.2.tif]

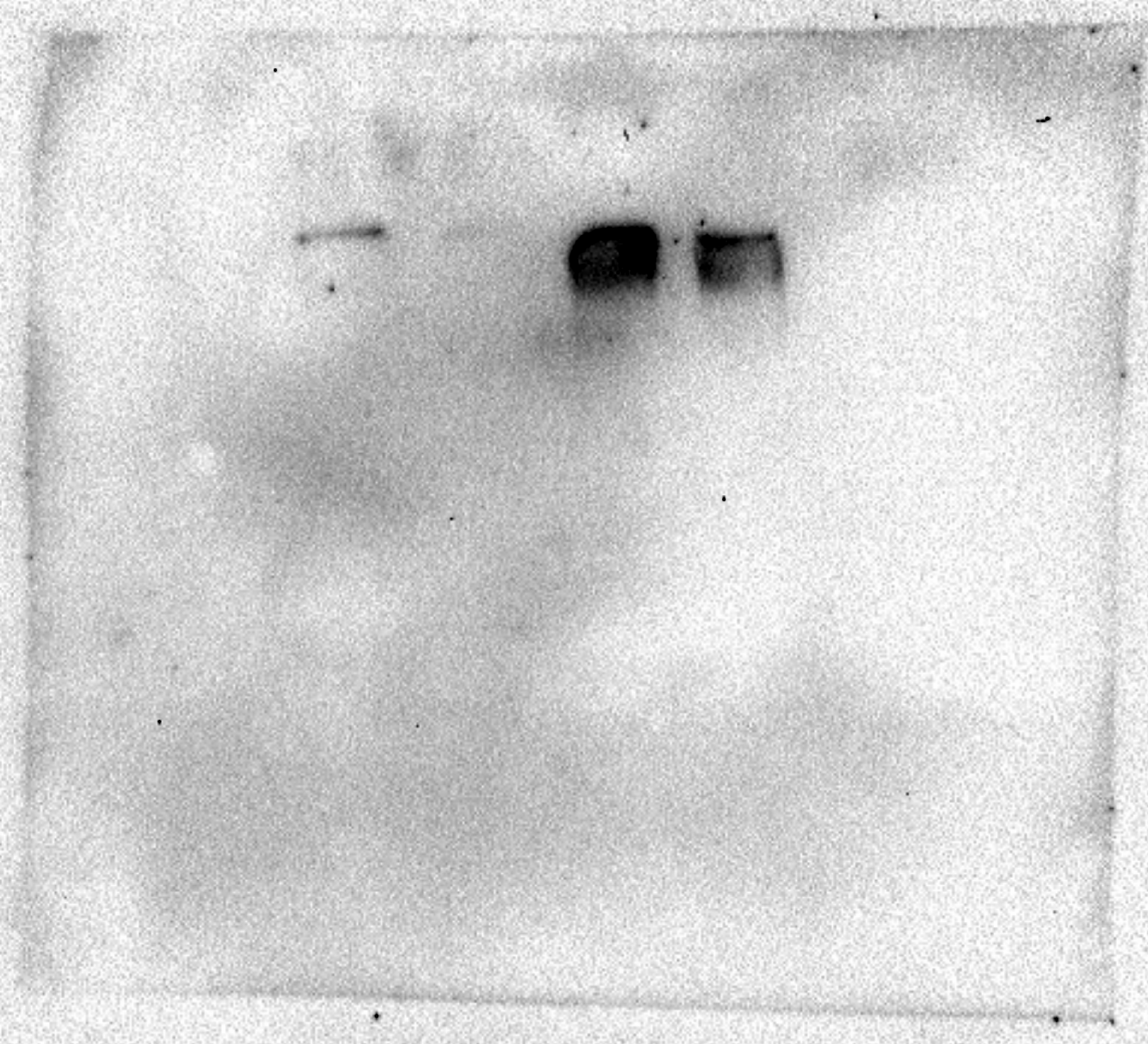

Supplement: Supplementary file 19 [file LSA-2019-00462_SdataF4.3.tif]

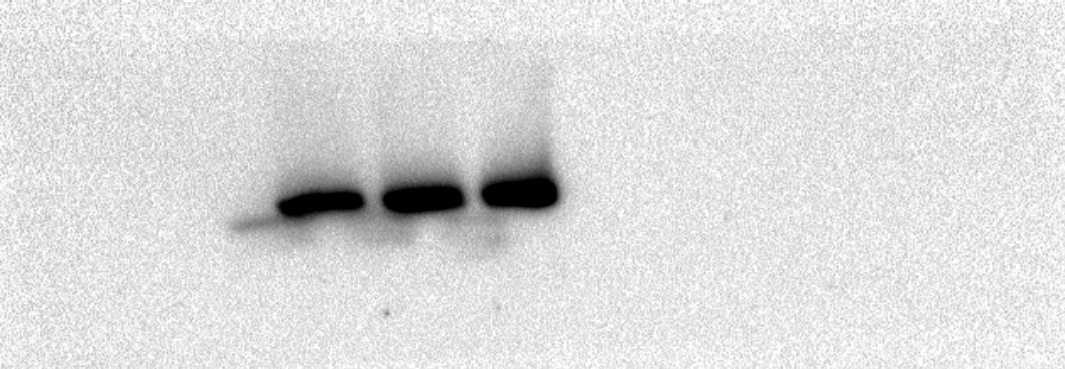

Supplement: Supplementary file 20 [file LSA-2019-00462_SdataF4.4.tif]

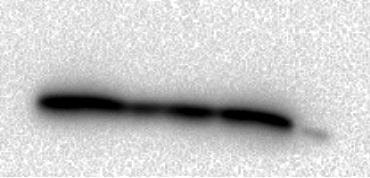

Supplement: Supplementary file 21 [file LSA-2019-00462_SdataF5.tif]

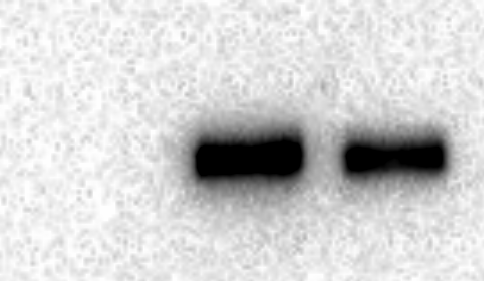

Supplement: Supplementary file 22 [file LSA-2019-00462_SdataF5.1.tif]

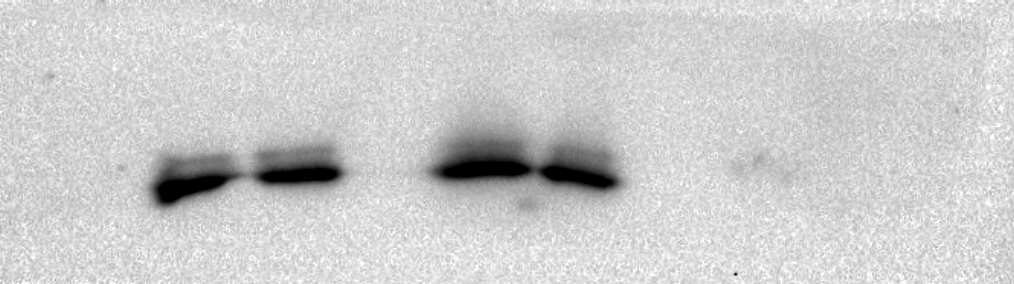

Supplement: Supplementary file 23 [file LSA-2019-00462_SdataF5.2.tif]

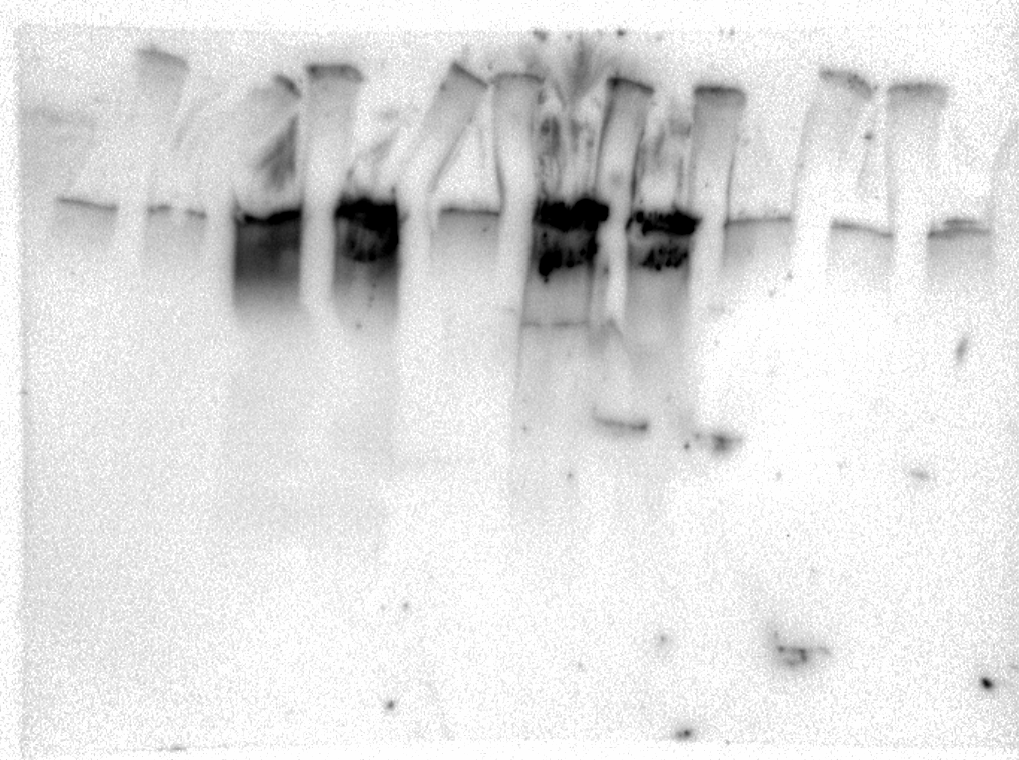

Supplement: Supplementary file 24 [file LSA-2019-00462_SdataF5.3.tif]

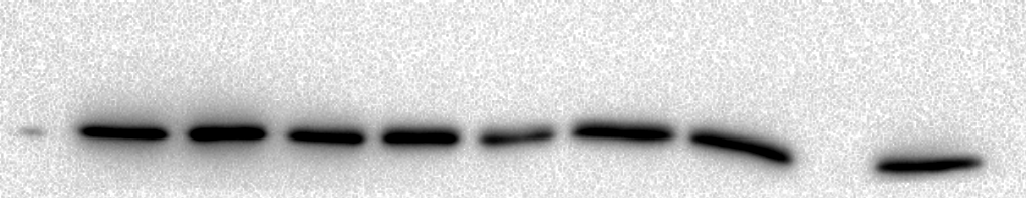

Supplement: Supplementary file 25 [file LSA-2019-00462_SdataF5.4.tif]

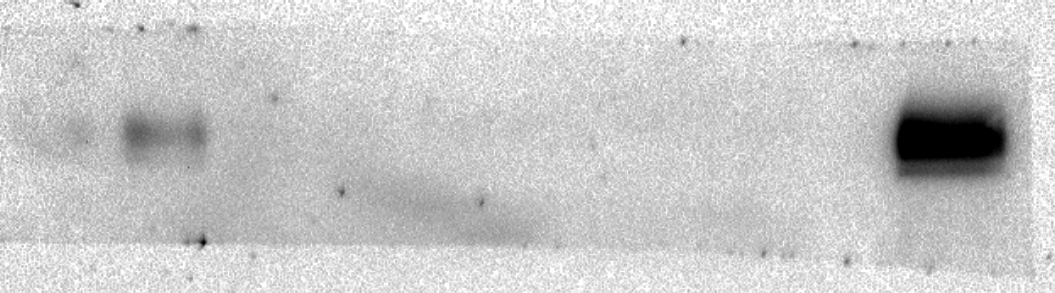

Supplement: Supplementary file 26 [file LSA-2019-00462_SdataF5.5.tif]

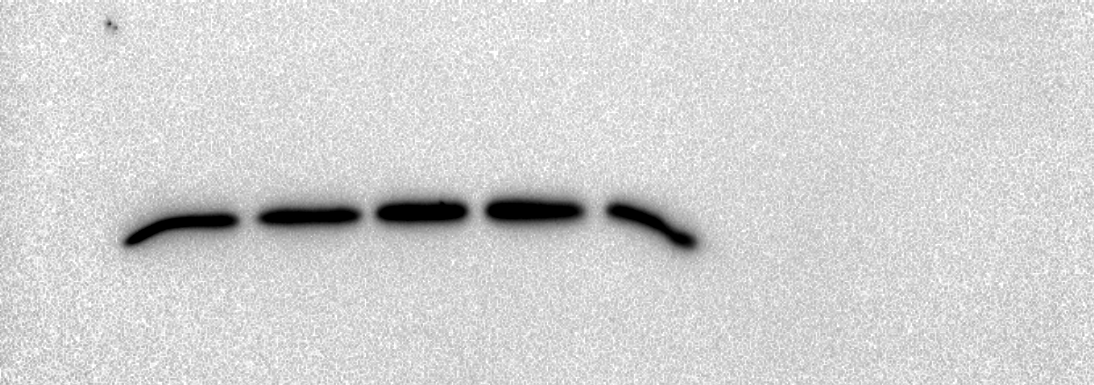

Supplement: Supplementary file 27 [file LSA-2019-00462_SdataF5.6.tif]

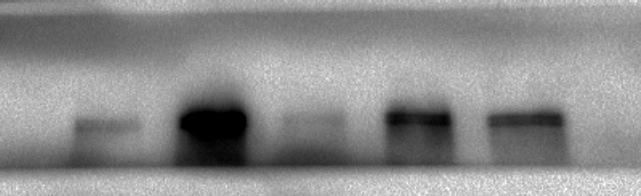

Supplement: Supplementary file 28 [file LSA-2019-00462_SdataF5.7.tif]

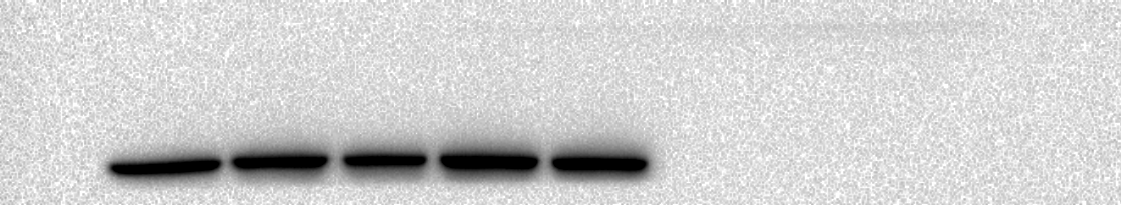

Supplement: Supplementary file 29 [file LSA-2019-00462_SdataF5.8.tif]

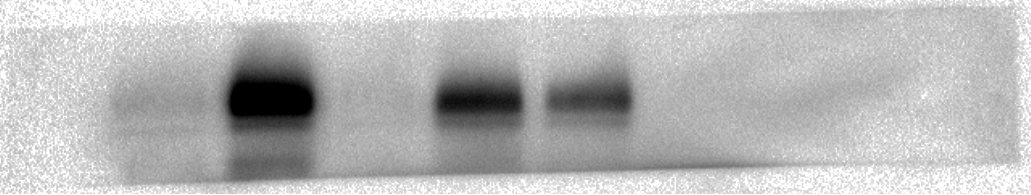

Supplement: Supplementary file 30 [file LSA-2019-00462_SdataF5.9.tif]

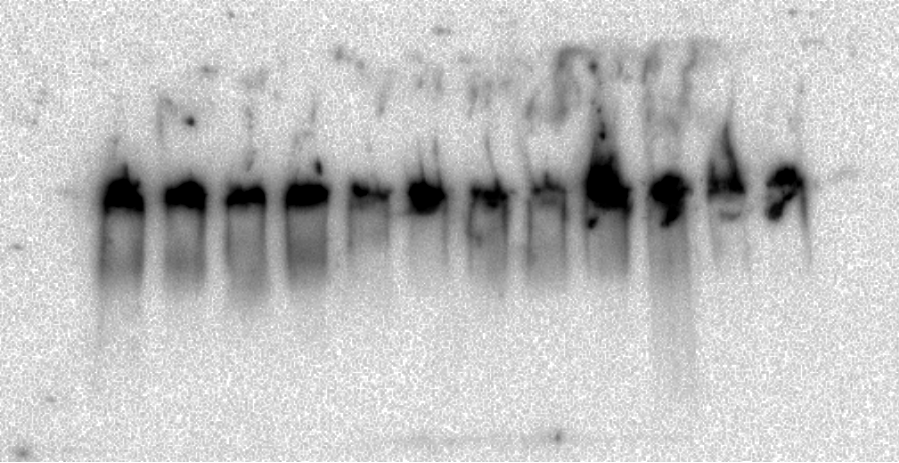

Supplement: Supplementary file 31 [file LSA-2019-00462_SdataF5.10.tif]

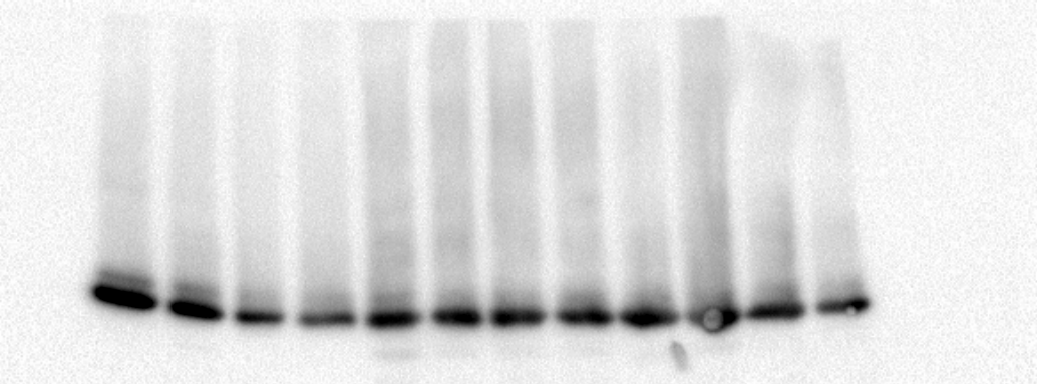

Supplement: Supplementary file 32 [file LSA-2019-00462_SdataF5.11.tif]

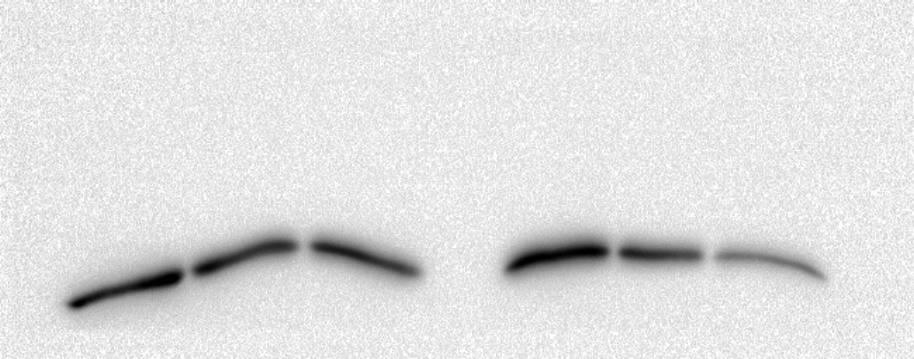

Supplement: Supplementary file 33 [file LSA-2019-00462_SdataF6.tif]

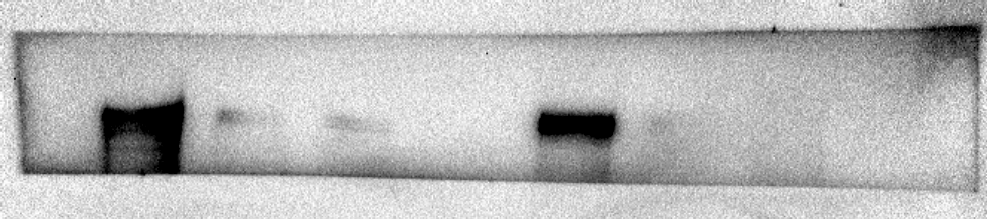

Supplement: Supplementary file 34 [file LSA-2019-00462_SdataF6.1.tif]

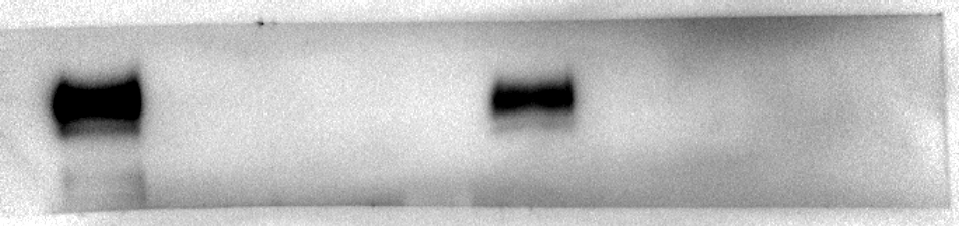

Supplement: Supplementary file 35 [file LSA-2019-00462_SdataF6.2.tif]

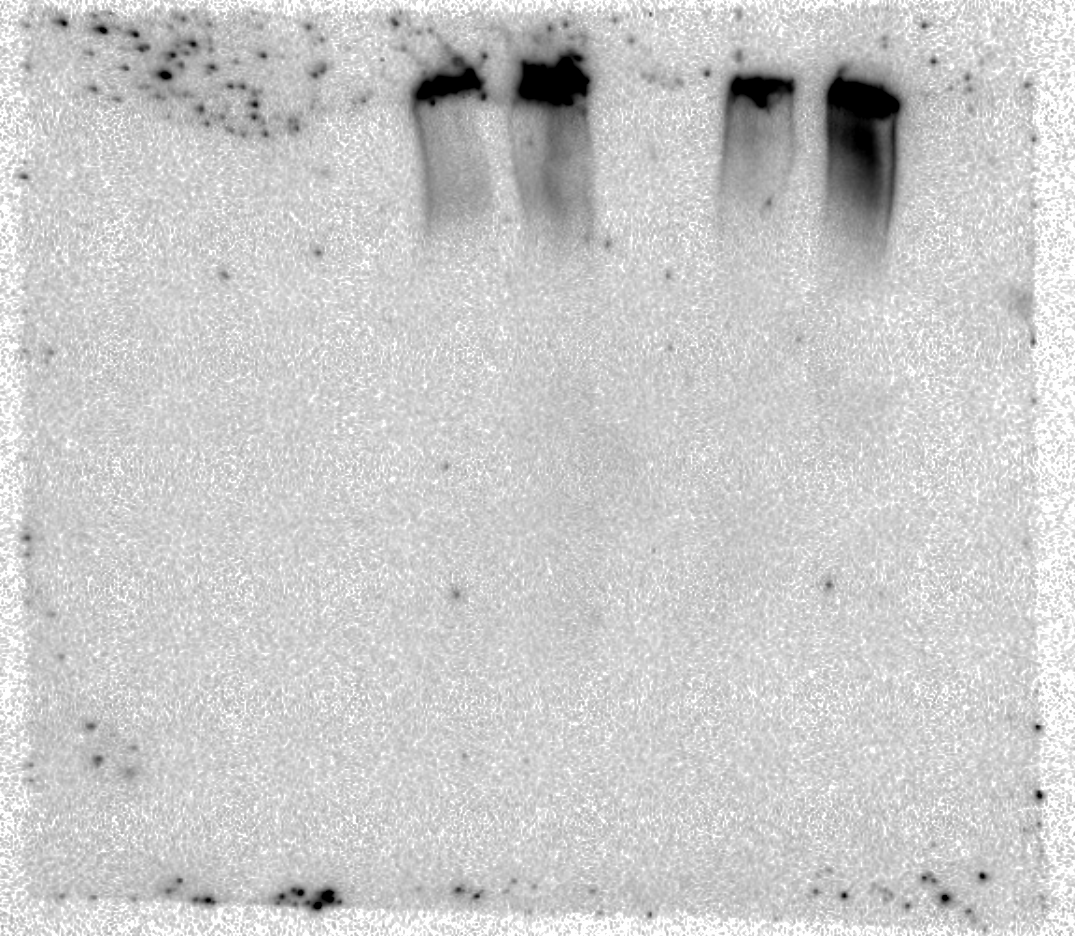

Supplement: Supplementary file 36 [file LSA-2019-00462_SdataF6.3.tif]

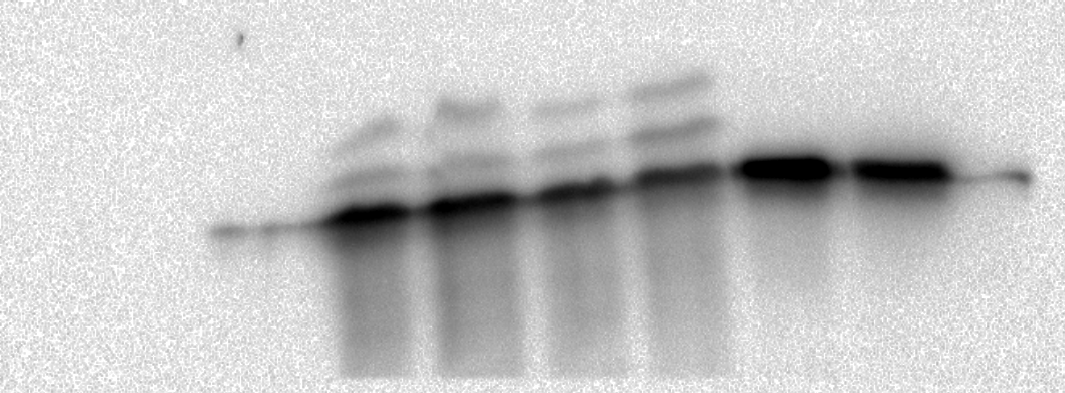

Supplement: Supplementary file 37 [file LSA-2019-00462_SdataF6.4.tif]
